# Costs of the Supervision, Performance Assessment and Recognition Strategy (SPARS) for improving medicines management in Nepal

**DOI:** 10.1080/20523211.2024.2421258

**Published:** 2024-11-18

**Authors:** Anika Ruisch, Ganesh Khatiwada, Anup Bastola, Christian Suharlim, Birna Trap

**Affiliations:** a Health Economics & Health Technology Assessment, Management Sciences for Health, Arlington, USAID Medicines, Technologies, and Pharmaceutical Services (MTaPS) Program, Arlington, VA, USA; b Management Sciences for Health, USAID Medicines, Technologies, and Pharmaceutical Services (MTaPS) Program, Kathmandu, Nepal; c Curative Service Division, Department of Health Services, Ministry of Health and Population, Kathmandu, Nepal

**Keywords:** Cost, medicine management, Nepal, performance assessment, public sector, essential medicines and health supplies

## Abstract

**Background:**

Nepal implemented a pilot of the Supervision, Performance Assessment, and Recognition Strategy (SPARS) program aimed to increase health workers’ ability to manage medicines through on- the-job training and support from a new cadre of Medicines Management Supervisors (MMS). This study aims to assess the implementation costs.

**Methods:**

Data from the SPARS central database and facilities was analysed to assess the total cost of the SPARS pilot including 293 public health facilities from 12 districts of 3 provinces, from May 2022 until July 2023. We estimated the number of health facilities to achieve a successful performance status, defined as a SPARS score ≥ 18.75 out of 25 (75%) and estimated the cost per facility to reach a successful score.

**Results:**

In total, 293 facilities received 838 visits, performed by 48 MMS for an estimated total cost of $226,531. 124 facilities (44.3%) reached a score of 18.75 points after two or more visits. On average, it costs $1827 USD per facility to reach a successful SPARS score.

**Conclusion:**

This study assesses the costs of implementing SPARS in 12 districts in Nepal. These findings can provide insights into further scaling up SPARS in Nepal or in other countries.

## Background

In 1975, the World Health Assembly endorsed the concept of essential medicines, triggering the adoption of essential medicines lists and the implementation of national drug policies in most low- and middle-income countries (LMICs) (Bigdeli et al., 2013). Efficient medicines supply; ensuring that essential medicines are affordable, available in appropriate doses, and with assured quality (NHRC, 2022), are integrally linked to strong health care systems. Medicines management processes include quantification, procuring, storage, stock management, prescribing, dispensing, ordering, and reporting. Adequate regulatory, financial, and human resources are crucial for the successful implementation of effective medicine management procedures, and facility staff with the necessary knowledge and skills are important actors (Oteba et al., 2018).

Nepal is committed to implementing Universal Health Coverage to guarantee that all citizens have fair access to high-quality medical care (NHRC, 2022). The Nepal Ministry of Health and Population (MOHP) recognises the need to strengthen its health care cadres’ capacity in medicines management and good pharmacy practices in government health facilities – ensuring medicines are available and accessible and are of safe, effective, and good quality and are used correctly (Bhusal et al., n.d.; Shrestha et al., n.d.).

To increase performance and build capacity in the management of medicines, a nationwide and comprehensive approach is required, as existing structures are decentralised, currently involving central, provincial, and local government. In 2015, Nepal adopted a new constitution (Oteba et al., 2018) and the country’s changed government structure has profoundly impacted the health sector’s administration and operations, including its supply chain and health logistics. Three levels of government in Nepal have been rights and obligations to help organise separate but coordinated actions in the health sector. The management of basic health care services is delegated to the municipal level by the constitution.

Over the years, health facility managers and medicine store staff have been trained in medicines supply chain management, and an electronic logistic management information system (e-LMIS) has been introduced in February 2018 with support from the US Agency for International Development (USAID)-funded Pharmaceutical Supply Management program (PSM). However, Nepal continues to face poor stock control, store management practices, dispensing practices, and quantification of needs; polypharmacy; a lack of or poorly maintained information management tools and records; are examples of the persisting challenges (Thapa et al., 2019).

Subcentral procurement processes under the new federalism system have led to a large degree of fluctuation in the cost of medications and there are not enough trained people at the local level (Thapa et al., 2019; The Medicines, Technologies, and Pharmaceutical Services (MTaPS) Program, 2022). The local government’s procurement and overall medicine management needs to be optimised in terms of forecasting, technical evaluation, and quality assurance for the procurement of necessary medications and medical commodities and stock and storage management.

### SPARS Nepal

SPARS offers a strategy to manage medicines and health supplies in five different domains: stock management, storage management, ordering and reporting, prescribing quality, and dispensing quality. The strategy combines supportive supervision to health facilities, with a performance assessment and a recognition strategy that has been found to improve medicines management and reporting quality in health facilities. SPARS uses practical performance indicators to flag areas for improvement in a real-life setting, guide and focus the supervision and provide the health staff with an understanding of their facility’s issues and achievements (Bhusal et al., n.d.; Shrestha et al., n.d.). SPARS was first developed based on evidence from national implementation in Uganda and the experiences of the Global Alliance for Vaccines and Immunization (Trap et al., 2016, 2018, 2020) and updated for Nepal. This the third in a series on the implementation of SPARS in Nepal, the other articles describe the methods and baseline results (Bhusal et al., n.d.; Shrestha et al., n.d.). To improve medicines management capacity at public health facilities, the Nepalese Curative Service Division (CSD), Department of Health Services MOHP, in collaboration with the USAID Medicines, Technologies, and Pharmaceutical Services (MTaPS) Program, piloted the supervision, performance assessment, and recognition strategy (SPARS) across 12 districts in 3 provinces in Nepal (Bhusal et al., n.d.).

The aim of this study is to estimate the costs of the SPARS pilot in Nepal’s public health facilities. We intended to estimate the annual cost of the SPARS pilot in Nepal and the costs per successful facility. This data are essential to determine further scale up in Nepal and for replication of SPARS in other countries.

## Methods

### Study setting

The MOHP and CSD purposely select three provinces out of seven for the pilot study based on population and health facility density and then randomly, by selecting notes with the eligible district’s names, selected four districts per province from districts situated in the lowland and hilly areas, but not mountainous areas, to ensure fair accessibility. SPARS was implemented in 293 public health facilities situated in the included 12 districts having a total of 838 supervisory visits, with support from USAID. 60 municipal health workers (e.g. pharmaceutical educated, nurses, clinical officers or midwives) were trained as medicines management supervisors (MMS). Each MMS selected three to nine health facilities to visit based on accessibility, convenience and need within their district to supervise regularly and monitor their performance (Bhusal et al., n.d.).

During the facility visits, the MMS complete the electronic data collection form and a paper based supervisory book with performance results. After finalising the assessment, MMS create a spider graph for each visit scoring each domain of the facility’s performance. The MMS identify areas that require improvement based on the evaluation, and following the assessment and during subsequent supervision visits, they assist facility staff in implementing change. After each visit, SPARS assessment data are uploaded into a centralised database developed based on the open-sourced Kobotoolbox^1^ to facilitate and manage evidence-based decision making.

The SPARS method and tool used in Nepal is a tool developed based on standardised drug management indicators and the World Health Organization rational drug use indicators (World Health Organization, 1993) that is described in detail in the SPARS Nepal method and baseline study (Bhusal et al., n.d.) and the first article of a three article series on its implementation in Uganda (Trap et al., 2016).

### Cost components of the SPARS pilot

We calculated the capital and operating costs to implement and run the SPARS pilot per facility between May 2022 to July 2023. The costs captured were those incurred to strengthen medicines management and are incremental to the costs of routinely supplying medicines in the health system. No costs of routinely supplying medicines are included, like salaries or supply chain related costs. An activity-based costing approach was used. We based our estimates on data from the SPARS intervention staff, programme reports, expenditure records, and invoices from May 2022 until July to identify activities and resource inputs used to implement SPARS. We collected data in both Nepalese Rupees (NRs) and United States dollars (USD) based on the currency used to procure inputs and implement activities. In the analysis and presentation of results, cost data are presented in USD conversions were calculated using a period average NRs 130.34: USD 1 (*Currency Converter, Foreign Exchange Rates, OANDA*, n.d.). We annualised all costs and capital costs were adjusted according to their expected useful life with an applied discount rate of 3% to obtain their annual depreciation value.

#### MMS training

For the SPARS Pilot, provincial, district and municipal management carefully selected five MMS in each district based on their management and communication skills and their interest in supply chain and the government’s medicine management issues and interest in becoming a supervisor. The district identified potential candidates who were willing to take on the MMS duties on top of their normal workload. Each district has a district MMS and four municipality MMS. We did not include the salaries for the health staff working as MMS as they are full-time government employees paid by the government and the MMS role was additional to their existing role and responsibilities.

MMS received a two-week (10 working days) theoretical training that includes medicine management, problem-solving, communication, mentoring, assessing performance using SPARS indicators, and work planning and reporting by faculty of Kathmandu University. The MMS received an additional one week (five working days) practical training in SPARS implementation, assessment, and reporting, followed by one day training implemented by the MTaPS technical advisors focused on challenging indicators. The costs of the three trainings are included as capital costs.

#### MMS equipment

To facilitate the performance assessment and ensure high-quality data, each MMS was provided with a computer, and a telephone to coordinate their visits and share the assessment data to the health facilities and submit the SPARS report to the SPARS database. MMS were provided with backpacks, carry bags, jackets, stationery, and a certificate for their performance.

Despite the possibility that computers and mobile devices could be utilised for reasons other than SPARS, we decided to take a conservative approach and include the entire cost of these capital goods. We estimated the costs of data management by including the cost of the MMSs’ computers, mobiles, and supervision costs.

#### Facility equipment

Health facilities received medicine management tools; standard treatment protocol, basic logistic training manual, national list of emergency medicine, national formulary, dispensing envelopes, dispensing logbook, cleaning & temperature log sheets, room thermometer, fridge thermometer, wall clock, carry bag, spray paint, calculator, and board markers. MMS were also provided with supervision tools for each facility, such as a SPARS assessment tool, and a supervisory book, as well as a white flex banner with a spider graph that displays and tracks each facility’s SPARS performance.

#### MMS supervision costs

MMS selected and assessed the performance of three to nine public health facilities every month, targeting all facilities in their area. Each of the sampled health facilities was supervised at least three times over the study period, while provided with targeted mentoring, supervision, and support in applying pharmaceutical management best practices. At the first visit, a baseline score was established, after which the first mentoring and supervision was given. The results of the first training were assessed at the next visit and so on (Bhusal et al., n.d.).

Expense payments per visit were paid as a lump sum to MMS. MMS receive Rs. 2600 (about $20 USD) when they submit a SPARS visit report to cover travel cost, data charges and food expenses for a residential and Rs. 6000 (about $46 USD) when supervising a remote facility that required 2 days including an overnight stay.

#### SPARS tools

Health facility staff were provided with medicines management tools to facilitate their work. Municipalities and health offices were provided with a certificate documenting their valuable support for implementation while provincial health logistics management centres, provincial health directorates, Management Division, and CSD officials receive a token of appreciation.

#### Programme management

Regular meetings support coordination and collaboration among the MMS, MTaPS technical advisors, municipalities, districts, provinces and CSD. Coordination took place at the following frequencies: bi-monthly meetings in the three provinces, bi-weekly coordination, and implementation meetings between CSD and MTaPS, and monthly virtual or face-to-face district level management and implementation meetings among the provincial and municipal focal persons and MMS. CSD collaborates with MTaPS technical advisors to prepare monthly SPARS reports and share them with MMS, districts, provinces, and central level stakeholders to show how supervision impacts medicine management performance. The cost of oversight and supervision of MMS by municipality, district, provincial and central level staff have been included. Costs for personnel to design, coordinate, and support to implement SPARS have been excluded as it is part of existing duties and salaries of the MTAPS Technical Advisors.

### Additional measurements

#### Measurement of intervention effectiveness

The purpose of SPARS is to improve medicine management at government health facilities. For this study, we used data from the SPARS central database and selected 293 facilities that started SPARS pilot implementation in May 2022 and followed each facility until July 2023 to assess SPARS effectiveness. When a health facility reached 75% of the maximum SPARS score 18.75 of 25 points during the three supervisory visits, its medicines management practices were deemed effective. The benchmark of 75% of SPARS score, that defined an acceptable medicines management (Trap et al., 2018) was based on agreement by MOHP and the MTaPS team and the same threshold for acceptability set in other countries implementing SPARS.

#### Measures of cost per successful facility

We divided the total incremental cost of SPARS implementation over the follow-up period (14 months for each of 284 facilities) by the number of facilities that attained the desirable SPARS score over the same period (124). We excluded 13 facilities that were visited less than two times.

#### Sensitivity analyses

To test the robustness of the unit cost and the variation of different cost drivers, we calculated the impact of 20% increase and decreases in cost in the major categories of the SPARS strategy: training of MMS, equipment for MMS, facility equipment and operating cost.

## Results

### Costs

Table 1 includes the costs of implementing SPARS, including approximately three visits to 293 facilities between May 2022 and July 2023. The 293 facilities received 838 visits, for an estimated total cost of $226,531 and an annualised cost of $194,168. Capital cost, including training and equipping the MMS to carry out SPARS was $182,763 (80.7%) and the operating cost was $43,768 (19.3%) including supervision, recognition items and programme management. Training (40.0%), laptops (24.4%) and supervision (12.8%) make up the largest share of the total costs. The costs include training of 60 MMS, in practice the work was done by 48 MMS as 12 MMS dropped out before the supervisory visits took place.

**Table 1.** Cost components of SPARS development and implementation including capital costs and operating costs.

Cost components		Total (NRS)	Total (USD)	Annual cost (USD 2022)
**Capital cost items**				
**MMS training**				
	2 weeks training	9,884,659	75,839	65,005
	1-week practical training	999,316	7667	6572
	Focused 1 day training	852,025	6537	5603
**MMS equipment**				
	Laptop with bag	7,200,558	55,246	47,353
	Mobile	2,099,999	16,112	13,810
	Printer (district)	560,000	4297	3683
**Facility equipment**				
	SPARS tools (supervision book, spider graph, cleaning log sheet, temperature log sheet, board marker, carry bag, spray paint)	803,153	6159	5282
	Standard Pharmacy equipment (dispensing envelope, bin card, dispensing log sheet, thermometer, fridge thermometer, job aids, wall clock, calculator)	1,421,140	10,904	9346
Subtotal		**23,820,850**	**182,763**	**156,654**
*(%) of total costs*		*(80.7%)*		

**Operating cost items**				
**MMS supervision costs**				
	Travel costs, data charges and food expenses incl overnight stay for remote facilities	3,779,210	28,996	24,853
**SPARS tools**				
	SPARS recognition items	140,250	1076	922
**Programme management**				
	Supervision by central, provincial, district and local level managers	31,500	242	207
	Coordination meetings	1,753,614	13,454	11,532
Sub total		**5,704,574**	**43,768**	**37,514**
*(%) of total costs*		*(19.3%)*		
**Total**		**29,525,424**	**226,531**	**194,168**

### Effectiveness of the SPARS pilot

Table 2 shows the effectiveness of the supervisory visits on stock management, storage management, ordering and reporting, prescribing quality, and dispensing quality during the 14-month pilot study period in Nepal. It represents the percentage increase in the number of facilities meeting effective standards of a 75% score or higher. The first visit established a baseline after which the facilities were trained. The progress was captured during follow-up visits after which another training session was provided.

**Table 2.** Effectiveness of SPARS in government health facilities reaching a score of ≥18.75 in Nepal.

Facility score	Baseline visit (%)	Last visit* (%)
Facilities with score <18.75	292 (99.7%)	155 (55.4%)
Facilities with score ≥18.75	1 (0.3%)	125 (44.6%)
Total SPARS facilities	293 (100.0%)	280 (95.6%**)
**Incremental effectiveness**		**124** (**44.3%)**

*Visit two or three after the baseline visit.

** Not 100% due to missed visits (facilities with <2 visits were excluded).

**Figure 1.**
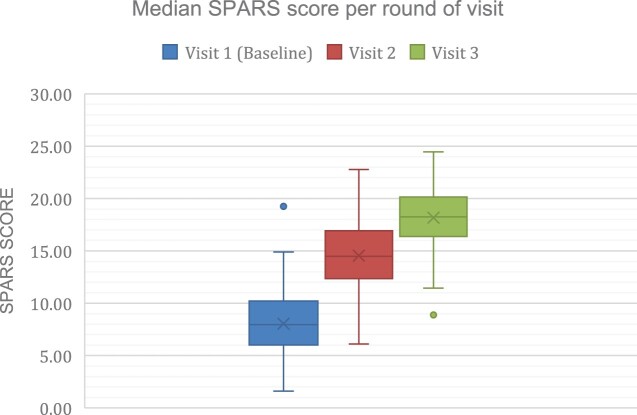
Illustrates the distribution of the health facility SPARS scores at visit 1 (baseline) to 3. *n* = 293.

At baseline, one health facility already achieved a score of ≥18.75. After the SPARS implementation, 124 facilities (44.3%) additional facilities achieved 75% or an 18.75 SPARS score or higher (Table 2). The average SPARS score at baseline was 8.04 [range 1.60–19.25]. The average SPARS scores increased to 14.56 [range 6.11–22.78] during visit 2 and to 18.17 [range 8.88–24.45] at visit 3. Of the 293 facilities in the SPARS pilot study, 265 facilities received all three visits, 15 received two visits and 13 health facilities received 1 visit. The data in this study reflect results to date. Missed visits in this dataset are because of the timing of this study during the ongoing SPARS programme, more visits are planned. The boxplot demonstrates the distribution of the scores and how health facilities reached higher median SPARS scores after each supervisory visit (Figure 1). The boxplot includes the mean, interquartile range, standard deviations, and outlier values of the SPARS scores for each round of visits.

### Cost per successful facility

Table 3 provides the cost of implementing the SPARS pilot study to the 293 facilities in Nepal including a total of 838 visits over 14 months. The investment of $226,531 over the 14 months successfully improved 124 facilities performance to achieve an adequate level of pharmaceutical management. It costs on average $1827 USD per successful facility. On average, a single supervisory visit in Nepal costs $270 USD. Visits to hard-to-reach facilities were more expensive as MMS were given a higher allowance to cover an overnight. A single supervisory visit to a residential facility is estimated to cost $264 whereas a remote visit costs $290. Our sample suggests that remote facilities have a higher chance of success (36 out of 69, 52.2%) and as such costs on average $1581 per successful facility. Conversely, only 89 out of 224 (39.7%) of residential facilities achieved an adequate SPARS score, costing on average $1928 per successful facility.

**Table 3.** Cost per successful facility.

	Residential	Remote	Total
Total number of facilities implementing SPARS	224	69	293
Total number of visits	642	196	838
Total number of facilities attaining/maintaining a score ≥ 18.75	89	36	125
Cost per facility implementing SPARS ($)	757	825	773
Cost per supervisory visit ($)	264	290	270
Cost per facility achieving SPARS score ≥ 18.75 ($)	1928	1581	1827
Total SPARS cost in USD ($)	**169,630**	**56,900**	**226,531**

### Sensitivity analysis

**Figure 2.**
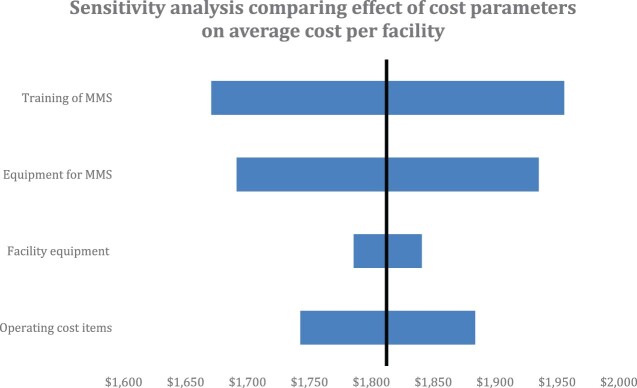
One-way sensitivity analysis comparing effect of variation of cost parameters on the average cost per facility achieving a successful SPARS score.

When we increase or decrease the cost of each of the categories by 20%, training and equipment have the highest impact on the cost per successful facility, reducing the cost of the training by 20% can bring down the cost of SPARS to $1668 per additional facility. Whereas the cost goes up to $1956 if training expenses increase by 20. In Figure 2 below, the blue bars represent how the listed cost categories (training of MMS, equipment for MMS, facility equipment and operating costs) impact the cost for each facility to reach a SPARS score of ≥18.75 in Nepal. The black line represents the incremental cost of $1827 USD calculated by this study.

## Discussion

This study estimates the cost of implementing a SPARS pilot in Nepal. Our study estimates the total cost associated with implementing SPARS, its cost components, as well as the cost per successful facility. Our study shows that 44.3% of facilities achieved an acceptable SPARS score after 2–3 visits. This is an interim result; the facilities will receive three or more additional visits that will be included in an effectiveness and impact assessment. SPARS contributes to strengthening supply chains by redistribution of near-to-expiry medicines, strengthening implementation health insurance schemes, introducing new guidelines and treatment regimen for non-communicable diseases (Trap et al., 2016, 2018, 2020). Improving supply chain management improves the access to quality medicines, which is important to ensure the optimal use of limited resources (Chukwu et al., 2017). The availability of medicines of good quality is the foundation for better health care for the people of Nepal and key to achieving universal health coverage.

Expanding SPARS to all 1,440 facilities in the sampled 12 districts would incur limited extra capital costs as it will utilise the same MMS team. We expect the additional costs to include all 1440 facilities to primarily consist of operating costs and facility equipment and would total $525,511 ($823.79 per successful facility – assuming a similar success rate). The capacity of the MMS can be shifted to new facilities within the district. Initially a facility needs frequent (monthly) visits by the MMS until it reaches an acceptable level or above, but thereafter, SPARS maintenance visits are less frequent, i.e. every 6 months, and eventually just once a year. Using the data and assumptions from this pilot, implementing, and rolling out SPARS nationwide would cost about $2.5 million USD or about 2% of the total government funding for essential medicines and supplies expenditures. A caveat of this projection is that SPARS implementation in other districts may have different characteristics and attrition of MMS than our study observed. Certain districts outside our sample are in more challenging geographic areas. A higher dropout rate of MMS would increase the overall budget, since training has the biggest impact on the overall budget as demonstrated in the sensitivity analysis. However, if trained health facility staff and MMS’s leave their present roles for other jobs, they can continue to apply their newly learned skills in new positions.

In our sample, residential facilities have a lower success rate and therefore a higher cost per successful facility compared to remote facilities. The explanation as to why remote facilities performed better following supervision is not known but could be linked to these remote facilities rarely receiving supervisory visits from the health management team and fewer patients per day, which allows them to become more engaged in improving their SPARS performance; including the additional attention and good mentoring to resolve identified issues. Additional visits or interventions to ensure effectiveness across all facilities in the included 12 districts would cost less as it would no longer require the expensive startup costs including training and laptops.

This pilot study solely focuses on assessing short-term measures and direct costs, highlighting the need to further examine the costs and benefits associated with indirect costs and long-term implementation and long-term viability. Other studies focused on the methods and baseline, and the improvement of SPARS indicators is described in a quasi-experimental pre–post study (Bhusal et al., n.d.; Shrestha et al., n.d.). The CSD and MTaPS team also assessed MMS’s inter-rater reliability of the SPARS indicators and based on that study, further training of the MMS was implemented to strengthen their mentor and supervisory capacity to build further facility capacity in the remaining time of the pilot study (Adhikari et al., n.d.). Studies from Uganda, where SPARS was implemented over several years, document SPARS long-term viability (Trap et al., 2016, 2018, 2020). It should be noted that at a minimum annual SPARS visit should be continued to all facilities to ensure sustainability of acceptable medicines management. Continued SPARS visits should be budgeted for and funded by the municipality and become an integrative part of the district health care budget to ensure long term viability.

The decision on whether an investment in SPARS is cost effective must reflect the health system context, challenges, and priorities (Leech et al., 2018). SPARS was funded by USAID and additional government funding, or donor support is needed to expand. Improving supply chain management improves access to quality medicines, which is important to ensure the optimal use of limited resources (Chukwu et al., 2017). The government of Nepal expenditures on health is currently USD 17 per capita per year with about 32% on pharmaceuticals and medical supplies (World Health Organization, 2020). To safeguard this significant investment and minimise waste, SPARS can provide a system foundation to manage scarce medicines resources well. While this study does not supply evidence on SPARS’ effect to reducing waste and expired medicines, other studies have shown its effectiveness in these domains (Seidman & Atun, 2017; Trap et al., 2018). Previously, SPARS boosted performance in important areas of medicine management in Uganda (Trap et al., 2018) and was found to have an effect beyond the scope of this study and the costing exercise. This costing study is limited as it does not provide answers in terms of the effect of the programme, further research on the cost effectiveness or a cost benefits analysis would provide more insight into the impact of SPARS in Nepal and would support further investment decisions.

### Strength and limitations

This study contributes to a growing body of evidence around the impact of the SPARS intervention. Strengths of this study are the buy-in from the Nepalese government, a large sample size, standardised assessment validated in other countries (Trap et al., 2016, 2018, 2020), and a multidimensional performance assessment. This study is not without limitations. Facilities were selected using a convenience sample, which will limit the representativeness of this study. Purposely selecting health facilities may have biased the sample, as MMS may have selected easy to reach and high performing facilities. The sample does not include any health facilities in the higher mountain regions of Nepal. Data were collected over 14 months and changes in scores may be results of confounding factors related to the five domains: stock management, storage management, ordering and reporting, prescribing quality, and dispensing quality. Moreover, MMS attrition and newly joined MMS may have influenced the scoring as well.

## Conclusions

This is the second study to document the costs of SPARS, currently through the pilot implemented in districts in Nepal, a multi-pronged intervention to improve medicines management practices at the facility level. In this pilot 44.3% (124/293) of the facilities reached a successful SPARS score after three visits demonstrating improved results in stock management, storage management, reporting and ordering, appropriate prescribing, and good dispensing practices. The 293 facilities received 838 visits, for an estimated total cost of $226,531 and an annualised cost of $151,142. On average, each successful facility required an investment of $1,827 USD. 73% of these costs are start-up costs, which is an investment the country can utilise as they expand SPARS. These findings can provide insight into the cost of further scaling up SPARS in Nepal and for replication of SPARS in other countries.

## List of abbreviations

**Table:** 

SPARS	Supervision, Performance Assessment and Recognition Strategy
MMS	Medicine Management Supervisors
MOHP	Ministry of Health and Population
CSD	Curative Service Division
MTaPS	Medicines, Technologies, and Pharmaceutical Services
NRs	Nepali Rupees
US	United States

## Data Availability

The data, data collection tool, analysis, and other materials are provided as part of the article or are available from the corresponding author.

## Footnotes

1 https://www.kobotoolbox.org/.
